# Perceptions and experiences on data sharing and linkage for research and the evaluation of public health policy.

**DOI:** 10.23889/ijpds.v7i3.2013

**Published:** 2022-08-25

**Authors:** Bethania de Araujo Almeida, Denise Moraes Pimenta, Mauricio Barreto

**Affiliations:** 1 Fiocruz - Center for Data and Knowledge Integration for Health (CIDACS)

## Objectives

This research seeks to understand viewpoints on the use of data containing personally identifiable information by a range of organizations for divergent purposes, focusing on the sharing and linkage of administrative data for the purposes of conducting research and evaluating public policies in Brazil.

## Approach

An exploratory approach was employed to perceive how the concepts relevant to this subject are understood, experienced and expressed by our interlocutors. A semi-structured interview technique was chosen to establish a base of scripted questions capable of building on and adapting to discussions with the participants, as well as addressing the questions that emerged during interviews. The interviewees were divided into three groups: data subjects (patients and beneficiaries of social welfare programs), researchers, and managers with experience in public policy in the areas of health and social protection. Specific scripts were elaborated for each group of respondents.

## Results

Groups and individuals are constantly balancing risks and benefits with regard to exposing and sharing their data; risks weigh more heavily depending on the individual’s socioeconomic context, which is permeated by intersectionality. The processing of personal data by the government raises more fears than actions taken by large technology companies. Individuals and social groups want to receive feedback on research carried out in their communities, and also desire to participate in the design of scientific research and the analysis of evidence used to guide public policies which directly affect them. Data governance is indispensable, requiring not only data management but also specific conditions on data sharing and linkage, principally regarding the sharing of administrative data aligned with legitimate public interests.

## Conclusion

Raising awareness and providing information on individual and collective rights on personal and sensitive data collection, as well as informing the public about the purposes of data sharing and linkage, is of utmost importance for responsible data management. Administrative data governance should be planned and implemented to foster trust and transparency among all involved and interested parties.

